# Research prospects in BioPsychoSocial medicine: new year reflections on the “Cross-Boarder Dialogue” paradigm

**DOI:** 10.1186/s13030-015-0038-0

**Published:** 2015-03-26

**Authors:** Florian Junne, Stephan Zipfel

**Affiliations:** Department Psychosomatic Medicine and Psychotherapy, Medical University Clinic Tuebingen, Medical University Hospital Tuebingen, Osianderstr. 5, 72076 Tuebingen, Germany; German College of Psychosomatic Medicine and Psychotherapy, Tuebingen, Germany

Looking back on an excellent year for the Journal of BioPsychoSocial Medicine, with numerous exciting papers addressing topics right at core of the Journal’s interest and often tackling issues at the forefront of international research trends, we first and foremost like to congratulate the Japanese Society of Psychosomatic Medicine on the performance of its official Journal in 2014!

Given the very special honor to contribute to the New Year’s Issue 2015 we would like to take the opportunity to reflect on a key paradigm of the Journal of BioPsychoSocial Medicine and the represented field of Psychosomatic Medicine: The imperative for cross-boarder dialogue and collaboration. One central aspect surely being the dialogue across the boarders of the biomedical and the psycho-social schools of thought, underpinned by the well supported hypothesis that the complex enigma of human existence can not be fully understood within the narrow boundaries of one or the other scientific community.

In the following we will discuss several selected perspectives on the dialogue and collaboration paradigm. First, we would like to discuss examples of recently published research related to the “stress-pandemic”. We then turn to the health-system dimension in underlining the importance of the trans-sectoral dialogue in psychosomatic medicine and BioPsychoSocial research, presenting examples of current studies on the treatment of anorexia nervosa (one of our own main clinical and research interests). Furthermore we would like to extend a very warm invitation to join us on the upcoming German Congress of Psychosomatic Medicine and Psychotherapy in March 2015 in Berlin; where we look forward to discuss the topic: ‘Psycho-Somatic, Dialogue Instead of Dualism’ with more then 1000 participants from all over the globe. We conclude in paying tribute to the Japanese-German friendship in the field of psychosomatic medicine, as a distinct example of a fruitful dialogue across the boarders of nations, cultures and continents.

## Societal dialogue: the BioPsychoSocial perspective on the “Stress-Pandemic”

Several manuscripts published in BioPsychoSocial Medicine in 2014 nicely illustrate the progress that was made during past decades in terms of integration of all three dimensions of the biopsychosocial model in clear-cut research designs. Kato et al. for example report in their pilot-study on healthy female nurses working in stressful environments and the association of their depressive symptoms with serum-levels of creatine kinase and lactate dehydrogenase [1]. They found the investigated biomarkers to be significantly associated with depressive symptoms in the analyzed sample. Whilst the nature of the findings is preliminary and indeed causation may not be inferred, the perspective of the hypotheses and the research design of the study may well be called “biopsychosocial”.

Such research is of importance especially in light of the increasing prevalence of stress-related diseases in most industrial nations. To investigate professional groups that are exposed to extraordinary levels of stress, including professions of the health work-force, may entail an important societal perspective as well. The latter in the sense that the resulting evidence may inform the societal debate and the political realm alike e.g. on the question how to create and regulate labour markets of the future and how to organize work place related health promotion and preventive measures [2].

We, the community of experts for psychosomatic medicine and biopsychosocial research, may here be asked to inform the public in an ongoing dialogue concerning questions such as: What levels of stress are tolerable for whom, depending on what set of factors of resilience? What biomarkers are suitable to predict and survey the burden of stress [3]? Which psycho-social interventions show the highest effectiveness to prevent or treat distress related psychosomatic diseases? What about quality of life and societal cost-consequences of distinct levels of stress for specific roles within certain industrial sectors? What is the evidence base of measures practiced by people who want to foster their individual stress-resilience or who want to reduce stress related symptoms?

The latter question e.g. was lately addressed in BioPsychoSocial Medicine by Yoshihara et al. who looked at a common trend in most industrialized nations with regards to self-administered enhancement of well-being and stress-resistance: Yoga. In their study, the team from the Department of Psychosomatic Medicine at Kyushu University, found significantly reduced stress-related symptoms after 12 weeks of regular yoga training in their sample [4]. The attempt to analyze such trends and to enrich the evidence base on practices such as Yoga could be followed more consequently in the field [5-7].

With regards to more specific stress-axis related diseases and complex (co-) morbidities, Hara et al. analyzed the effects of gender, age, family support and treatment on perceived stress and the coping of patients with type-2 diabetes [8]. The study found significant gender effects on perceived stress, coping and diet regimen in the investigated sample of patients with type-2 diabetes. Age had an effect only in males, whilst in women the “psychological impact of diabetes” was higher than in males. The study-team concludes that on the basis of their findings, individualized stress and coping related interventions for affected patients can be facilitated [8,9].

The study by Hara et al. thereby clearly refers to the concept of “tailored interventions” which takes into account the pattern of individual characteristics and needs of patients when planning and conducting interventions [8]. Besides being based on sound ethical grounds, the tailored interventions approach promises more precise and hence – ceteris paribus – more effective interventions. To enable tailored interventions in a cost-effective manner we do need to stay focused on the development of specific instruments to detect relevant patterns of patient characteristics to adjust our interventions accordingly. Saigo et al. for example, have contributed to this area of psychometrics and psycho-diagnostic in validating the Japanese version of the visceral sensitivity index for IBS patients in a group of Japanese University students [10]. According to the authors, the validated new version of the instrument may now enable e.g. the cross-cultural comparison of psychological aspects of IBS. In practical terms however, such well designed diagnostic instruments do substantially enrich the most important dialogue we can engage in - the dialogue with our individual patient [11,12].

## Cross-sector dialogue - the health system perspective

With regards to the health system perspective, experts from the field of Psychosomatic Medicine are well aware of the unique importance of settings and conceptual modalities in the treatment of psychosomatic diseases. Psychosomatic entities are (in their onset and during the course of treatment) decisively determined by the complex interplay of individual patient characteristics, psycho-social determinants and conceptual treatment structures [13]. Hence, it is evident that treatment settings, especially the health system sectors (i.e. ambulatory, day-clinic or inpatient sector) have significantly different roles to play or functions to fulfill along the care-continuum. The dialogue across the treatment sectors can thereby be seen to be of significant importance not only from a clinical but from a health systems research perspective as well.

Indeed health service structures and practices differ widely across countries. Nevertheless, there might be international consensus concerning the view that the choice of the sector where in a patient needs to be treated primarily depends on symptom severity and on the degree of physical impairment. However, what do we know about the plethora of potential other characteristics that might mediate or moderate treatment success as a function of treatment-sector?

Kawai et al. shed some light on this important issue in asking what psychosocial factors are associated with immediate (urgent) inpatient admission of patients suffering from anorexia nervosa [14]. The group analyzed a total sample of 133 patients that were either urgently admitted or scheduled for planned admission. The results of this beautiful health services research study, primarily confirm that BMI and the associated physical condition are the main drivers for immediate inpatient admission at the study-centre. None of the hypothesized psychosocial factors showed relevant associations with the decision for one or the other patient pathway. Kawai et al. also showed that the urgently (because of lower BMI) admitted group had poorer outcomes in terms of psycho-social adjustment two years after inpatient treatment. Given this finding one could ask whether e.g. a step-down approach for the severely-ill patient group might be advisable, where patients receive day-patient care following intense inpatient treatment to facilitate the setting transfer from inpatient to ambulatory care [14].

The latter question points to the seminal study on the efficacy of day-patient treatment for adolescent patients with anorexia nervosa conducted by Herpertz-Dahlmann and colleagues [15]. The study investigated whether day-patient treatment for adolescent non-chronic anorexia nervosa patients is inferior to inpatient treatment with regards to BMI at 12-months follow-up. The multi-centre study was designed as a randomized, open-label, non-inferiority trial. Patients admitted to the study-protocol underwent either inpatient or day-patient treatment following a standardized three-week inpatient period [15]. The results show that the day-patient setting was no less successful in the treatment of patients with anorexia nervosa when measured in BMI at the 12-months follow up. In absolute values BMI after the day-patient treatment tended to be higher than in the inpatient group. The day-patient setting thereby was associated with an average of 20% cost-savings (insurer perspective) at the time of discharge [15]. Hence, in cost-efficacy terms the day-patient setting was superior to the inpatient pathway in this study. Such evidence may well justify an intensified dialogue across health system sectors and it calls for further research on the issue of optimal trans-sectoral patient pathways including e.g. criteria dependent stepped-care procedures with evidence based dosages.

Once a patient is transferred to the ambulatory sector the question may arise, what exact treatment is to offer to a patient with anorexia nervosa? The ANTOP-study (Zipfel et al.) sought to answer such questions in comparing Focal Psychodynamic Therapy (FPT) with Cognitive Behavioral Therapy (CBT) against optimized treatment as usual (TAU-O) in the treatment of adult patients suffering from anorexia nervosa [16]. The randomized, controlled, multi-center trial showed that FPT was the most efficacious psychotherapeutic strategy when looking at BMI at 12-months follow-up. CBT however showed quicker results (increase in BMI) in earlier treatment phases [16]. Beside the fact that the control-arm (TAU-O) proved to be a solid basic strategy, we might learn from the ANTOP-study that 1) specialized (manualised) psychotherapeutic strategies have once again proofed to be the gold-standard for the psychosomatic and psychotherapeutic profession and 2) integrative approaches with combinations of techniques from different psychotherapeutic schools (e.g. FPT and CBT) are indeed a very promising agenda of evidence based psychosomatic medicine if we seek to optimize results for our patients. Here again our inclination to dialogue may flourish in fostering the collaboration among different psychotherapeutic schools and traditions.

## Cross-journal dialogue: joint publication initiative on eating disorders

Given the various open questions concerning the nature and evolution of eating disorders and the ever increasing scientific efforts to understand the related phenomena, we very much welcome the first joint publication initiative of BioPsychoSocial Medicine and the Journal of Eating Disorders on this matter. Themed “Current Status of Eating Disorders: General and Special Population Studies” the cross-journal thematic series called for research articles or systematic reviews that “inform on the prevalence, incidence, risk factors, morbidity and other associated factors that have implications for primary, secondary or tertiary prevention” of any type of eating disorder. We ourselves look forward to the publication of this first joint thematic series early in 2015 and kindly recommend the issue to the similarly intrigued colleague.

## Congress of dialogue: the German congress of psychosomatic medicine

Psycho – somatic, Dialogue instead of Dualism” – is the motto 2015 of the nationally and internationally recognized German Congress of Psychosomatic Medicine and Psychotherapy. With the emphasis on “dialogue” the 2015 annual conference of the German College of Psychosomatic Medicine and Psychotherapy and the German Society of Psychosomatic Medicine, focuses on the dialogical nature of mind and body as well as on the dialogue with colleagues from other medical disciplines and e.g. renowned representatives of the humanities.

For 2015 the congress president is very pleased to host (amongst others) renowned international key-note speakers such as: Niall Boyce (Editor in Chief, Lancet Psychiatry), Giovanni Fava (Editor in Chief, Psychotherapy and Psychosomatics), Ulrike Schmidt (King’s College London), Michael Sharpe (Oxford), Louis G. Castonguay (Pennsylvania State University) and Peter White (London). Furthermore, we will run research methodology oriented courses in English language to facilitate participation of our international guests. This track e.g. includes the high-profile Carus-Master-Classes in research methodology. The latter format will be held for the first time in 2015 and is subject to special application (see the English web-site of the congress for further information). International applications are very welcome for this format! Not only for the Carus-Master-Classes but for the 2015 congress at large we invite all colleagues from Japan and the Asia-Pacific Region very warmly!

## Cross-continental dialogue: the Japanese-German friendship in psychosomatic medicine

Close relationships between the Japanese and the German Medical Community date back at least to the 19^th^ century when Erwin Bälz (who studied medicine at Tuebingen University), served between 1876 and 1905 as the personal physician to his Majesty the Emperor of Japan Meiji Tenno Mutsuhito. More than hundred years later, we can rejoice in the fact that especially in the field of Psychosomatic Medicine our Societies and their members engage in an ongoing fruitful exchange about current trends and challenges in research and practice of psychosomatic medicine! Beside the joint initiatives by Prof. Ikemi and Prof. Schepank in the 1980s, the close cross-continental connection was expressed e.g. by an official agreement on collaboration signed in the context of the 16^th^ Annual Conference of the Society of Psychosomatic Internal Medicine in 2011.

Each year delegates from the Psychosomatic-Societies of both countries contribute to the major conferences of either side. For 2015 we do look forward for example to the scientific symposium organized by our Japanese colleagues at the German Congress of Psychosomatic Medicine and Psychotherapy in March 2015 in Berlin. For this congress we also aim to conduct a Japanese-German Case Conference as a new special format. Conversely, delegates from e.g. the German College of Psychosomatic Medicine took part in the Congress of the Japanese Society of Psychosomatic Internal Medicine in November 2014 and colleagues from Germany aim to present their latest insights at the 56^th^ Congress of the Japanese Society of Psychosomatic Medicine in June 2015 in Tokyo.

As Chiharu Kubo has put it in his New Year Address 2013: To “…build strong bonds with individual researchers and clinicians” [across the boarders of nations] ultimately will also “…help us provide the best possible care to our patients” [17].

On behalf of the German College of Psychosomatic Medicine and the German Society of Psychosomatic Medicine, we would like to express our deep gratitude for the enriching personal and professional encounters our delegates have made in past years when sharing our views on the field of psychosomatic medicine and psychotherapy with colleagues from Japan. May this excellent example of a cross-continental dialogue prosper in 2015 and beyond in the best interest of both sides!

フローエス ノイエス ヤー 2015!
